# Huntingtin Acts Non Cell-Autonomously on Hippocampal Neurogenesis and Controls Anxiety-Related Behaviors in Adult Mouse

**DOI:** 10.1371/journal.pone.0073902

**Published:** 2013-09-03

**Authors:** Patrick Pla, Sophie Orvoen, Caroline Benstaali, Sophie Dodier, Alain M. Gardier, Denis J. David, Sandrine Humbert, Frédéric Saudou

**Affiliations:** 1 Institut Curie, Orsay, France; 2 CNRS UMR3306, Orsay, France; 3 INSERM U1005, Orsay, France; 4 University Paris-Sud, Orsay, France; 5 EA3544, Faculté de pharmacie, University Paris-Sud, Châtenay-Malabry, France; Emory University, United States of America

## Abstract

Huntington’s disease (HD) is a fatal neurodegenerative disease, characterized by motor defects and psychiatric symptoms, including mood disorders such as anxiety and depression. HD is caused by an abnormal polyglutamine (polyQ) expansion in the huntingtin (HTT) protein. The development and analysis of various mouse models that express pathogenic polyQ-HTT revealed a link between mutant HTT and the development of anxio-depressive behaviors and various hippocampal neurogenesis defects. However, it is unclear whether such phenotype is linked to alteration of HTT wild-type function in adults. Here, we report the analysis of a new mouse model in which HTT is inducibly deleted from adult mature cortical and hippocampal neurons using the CreER^T2^/Lox system. These mice present defects in both the survival and the dendritic arborization of hippocampal newborn neurons. Our data suggest that these non-cell autonomous effects are linked to defects in both BDNF transport and release upon HTT silencing in hippocampal neurons, and in BDNF/TrkB signaling. The controlled deletion of HTT also had anxiogenic-like effects. Our results implicate endogenous wild-type HTT in adult hippocampal neurogenesis and in the control of mood disorders.

## Introduction

Huntington’s disease (HD) is a fatal neurodegenerative disorder with motor, anxio-depressive and cognitive components. It is caused by a dominantly inherited mutant allele of the gene encoding huntingtin protein (HTT) in which the number of CAG triplet repeats, encoding glutamines, in the N-terminal part of the protein exceeds 36. Onset is generally in midlife and the disease leads to death within 15-20 years during which motor control and cognitive functions decline. Anxiety and depression are prevalent among HD patients, and often appear earlier than the motor symptoms [1,2].

Various mouse lines have been generated to study the etiology of HD symptoms [3,4]. Some are transgenic mice overexpressing N-terminal parts of HTT or the full-length HTT protein with an expanded polyQ stretch; other are knock-in mice in which the wild-type polyQ repeat has been replaced by an expanded repeat. In most of these models, anxio-depressive related behaviors develop before the onset of motor symptoms [5,6]. Most of these models also show altered neurogenesis in the hippocampal dentate gyrus (DG) involving abnormal proliferation, survival or maturation [3,5].

Extensive analyses of these mice have revealed toxic effects of the pathogenic form of HTT. However, it is unclear whether the neurological and behavioral defects observed in these mice are related to abnormal function of wild-type HTT or new functions associated with the polyQ expansion. The recent development of a knock-in mouse strain in which Cdk5 phosphorylation sites in HTT protein are mutated and thus cannot be phosphorylated showed enhanced hippocampal neurogenesis associated with a reduction of anxiety/depressive-like behaviors, thus linking HTT function and the control of mood disorders [7]. However, the contribution of adult-related mechanisms to neurogenesis remains to be determined as these various mouse strains express mutant HTT from early developmental stages. In addition, these mice express also the mutation in all neurons (mature and newborn) making impossible to study specifically non-cell autonomous effects on neurogenesis. We therefore generated a conditional knockout of HTT in adult mice using an inducible CreER^T2^/Lox system activated by tamoxifen injection. The promoter used, the *CamKIIα* promoter, targets the expression of CreER^T2^ to mature cortical and hippocampal neurons in the chosen transgenic line [8]. Neural stem cells or immature neurons that do not express CamKIIα at the time of tamoxifen injection maintain HTT expression. Consequently, this model allows us to address the consequences of HTT deletion only in adult neurons on the development of newborn neurons. We analyzed the consequences of HTT deletion 6 months after tamoxifen injection. Indeed, in most previous studies, expression of mutant *Htt* or deletion of endogenous *Htt* either in the forebrain or in the projecting neurons from the cortex, result in slowly progressing phenotypes and only in aged mice [9–11]. We report that HTT depletion from hippocampal neurons caused non-cell autonomous defects of survival and dendritogenesis in newborn neurons. We observed a decrease in BDNF transport and release thereby reducing BDNF/TrkB signaling in mutant hippocampus. Mutant mice also displayed abnormal anxiety-related behavior. These findings suggest that wild-type HTT in mice, by controlling BDNF dynamics and its downstream signaling in the mature hippocampus, has a prominent role in hippocampal neurogenesis in adults, and regulates anxiety-related behavior.

## Results

### Inducible depletion of huntingtin in hippocampus and cortex

**Figure 1:**
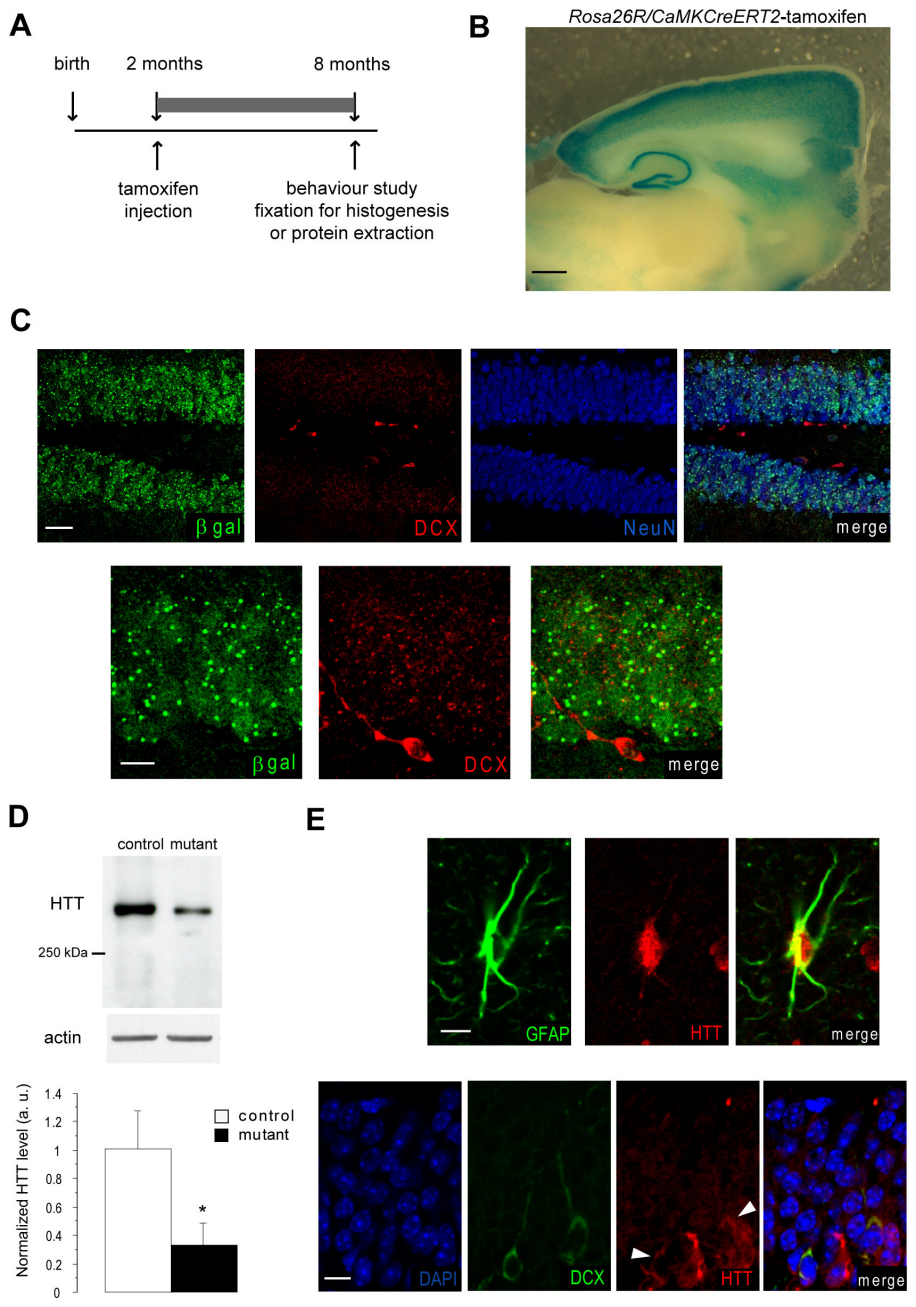
Tamoxifen injection in *CaMKCreER* ^*T2*^ mice activates Cre expression in mature but not newborn hippocampal neurons. (**A**) Design of experiments performed on *CaMKCreER* ^*T2*^ *;ROSA26R, CaMKCreER* ^*T2*^ *; Hdh* ^*flox/flox*^ and *WT; Hdh* ^flox/flox^ mice. (**B**) Whole mount X-gal staining of a parasagittal brain section of a *CaMKCreER* ^*T2*^ *;ROSA26R* mouse injected with tamoxifen. Scale bar: 500 µm. (**C**) Immunofluorescence in the hippocampal region of tamoxifen-injected *CaMKCreER* ^*T2*^ *;ROSA26R* mouse with antibodies recognizing β-galactosidase, DCX and NeuN. Scale bar for upper panel: 30 µm ; scale bar for lower panel: 10 µm. (**D**) Representative Western-blot of hippocampal proteins extracted from mutant and control mice 6 months after tamoxifen injection and incubated with anti-Htt (4C8) and anti-actin antibodies. Data are the mean +/- SEM of the ratios Htt/actin, normalized so that the mean value of controls is equal to 1 (n= 4-5 per group). * p<0.05 (**E**) Hippocampi of mutant mice 6 months after tamoxifen injection were co-immunostained with antibodies recognizing GFAP or DCX and HTT (4C8). White arrows indicate HTT expression in DCX positive neurons. Scale bar for upper panel : 10 µm; scale bar for lower panel : 8 µm.

To generate mice deleted for HTT in the adult brain, we first crossed *CaMKCreER* ^*T2*^ mice with *ROSA26R* mice to verify that the Cre recombinase is activated only in mature cortical and hippocampal neurons in the forebrain. Two-month old *ROSA26R;CaMKCreER* ^*T2*^ mice were injected with tamoxifen, and their brains were harvested and fixed 6 months later (Figure 1A). Whole-mount X-gal staining gave results similar to those of previous studies [8], including notably a strong induction of the beta-galactosidase reporter activity in the cortex and hippocampus (Figure 1B). Immunofluorescence staining in the DG of the hippocampus revealed beta-galactosidase expression in most NeuN-expressing mature neurons but not in DCX-expressing immature neurons (Figure 1C). Hence, these transgenic mice can be used to delete floxed genes specifically from mature hippocampal neurons without affecting the genotype of newborn neurons.

We then crossed *CaMKCreER* ^*T2*^mice with *Htt* ^*flox/flox*^ mice to obtain *CaMKCreER* ^*T2*^ *; Htt* ^*flox/flox*^ mice referred to throughout the text as mutants, and their *WT; Htt* ^flox/flox^ littermates that were used as controls. Tamoxifen was injected when mice were 2 months old, and the consequences of HTT deletion were studied 6 months later. HTT was substantially less abundant in protein extracts from mutant hippocampi than in protein extracts from control hippocampi (Figure 1D). Expression of HTT was not completely abolished, because HTT was still expressed in astrocytes labeled with GFAP or newborn neurons expressing DCX (Figure 1E).

### Huntingtin depletion in mature cortical and hippocampal neurons affects survival and maturation of new-born neurons

Hippocampal neurogenesis in the adult can be evaluated by analyzing the proliferation, survival and differentiation/maturation in the dentate gyrus (DG) in the hippocampus. These steps have been shown to be affected in various mouse HD models, suggesting that loss of HTT may also affect neurogenesis [3].

**Figure 2:**
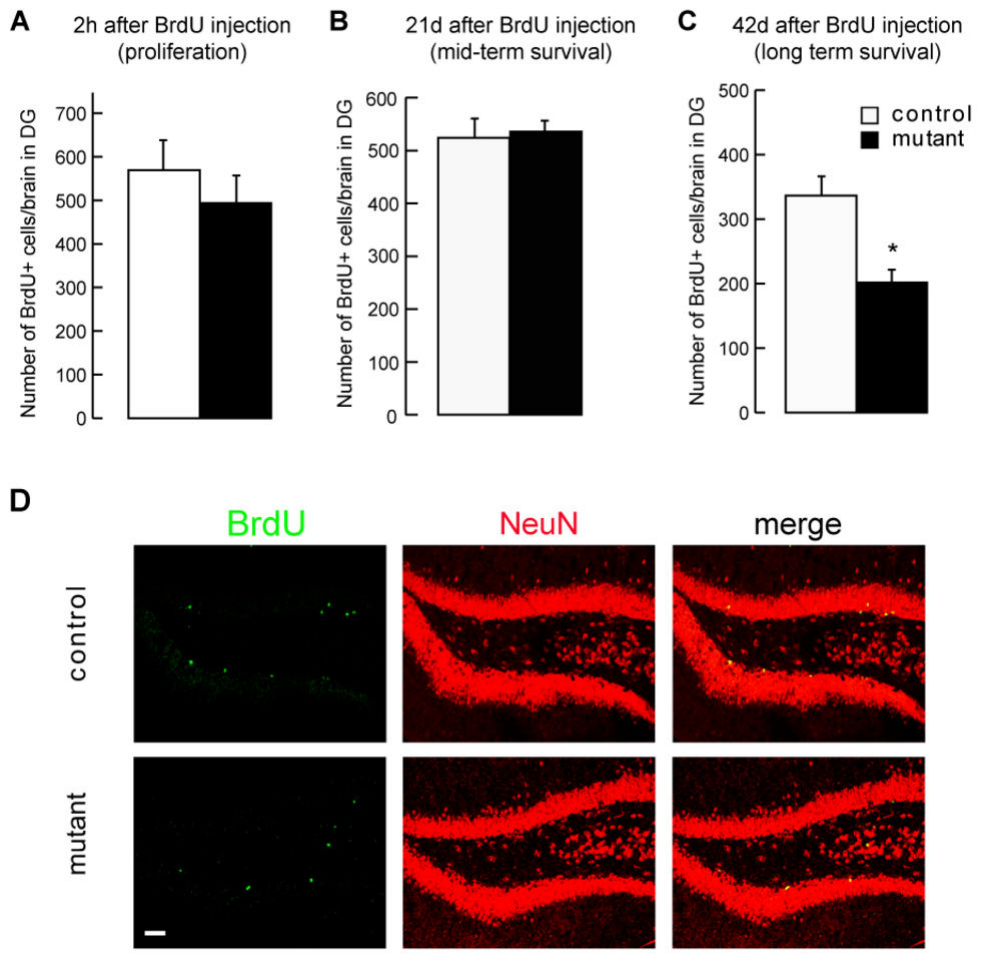
Deletion of huntingtin in mature neurons alters survival of newborn hippocampal neurons. All mice were injected with tamoxifen 6 months before sacrifice. (**A**) BrdU was injected 2 h before sacrifice into mutant and control mice. Immunofluorescence was performed with an anti-BrdU antibody. Data are the mean +/- SEM of the positive BrdU positive cell counts in DG from 3–4 brains per group. (**B**) BrdU was injected 21 days before sacrifice into mutant and control mice. Immunofluorescence was performed as in A. Data are the mean +/- SEM of the positive BrdU positive cell counts in DG from 3 brains per group. (**C**) BrdU was injected 42 days before sacrifice into mutant and control mice. Immunofluorescence was performed as in A. Data are the mean +/- SEM of the positive BrdU positive cell counts in DG from 3–4 brains per group. * p<0.05 ** p<0.01. (**D**) BrdU (green) and NeuN (red) immunofluorescence in DG of control and mutant mice injected with BrdU 42 days before sacrifice into mutant and control mice. Scale bar : 50 µm.

To analyze proliferation, we injected BrdU 2 hours before sacrificing control and mutant mice. The numbers of BrdU-positive neurons in the DG did not differ significantly between control and mutant mice (Figure 2A).

We next analyzed mid-term and long-term survival of newborn hippocampal neurons by injecting BrdU into control and mutant mice and analyzing the numbers of BrdU-immunopositive neurons 21 days and 42 days after injection. We observed no significant difference in the survival of neurons in the medium term (21 days; Figure 2B), but we found a significantly poorer long-term survival of neurons in the DG of mutant mice (Figure 2C-D). The total numbers of DCX^+^ neurons in mutant and control mice were not significantly different (Figure S1) These experiments show that the long-term survival of newborn DG neurons is affected non-cell autonomously when HTT is depleted in mature cortical and hippocampal neurons.

### Huntingtin depletion reduces dendritic arborization of hippocampal neurons

**Figure 3:**
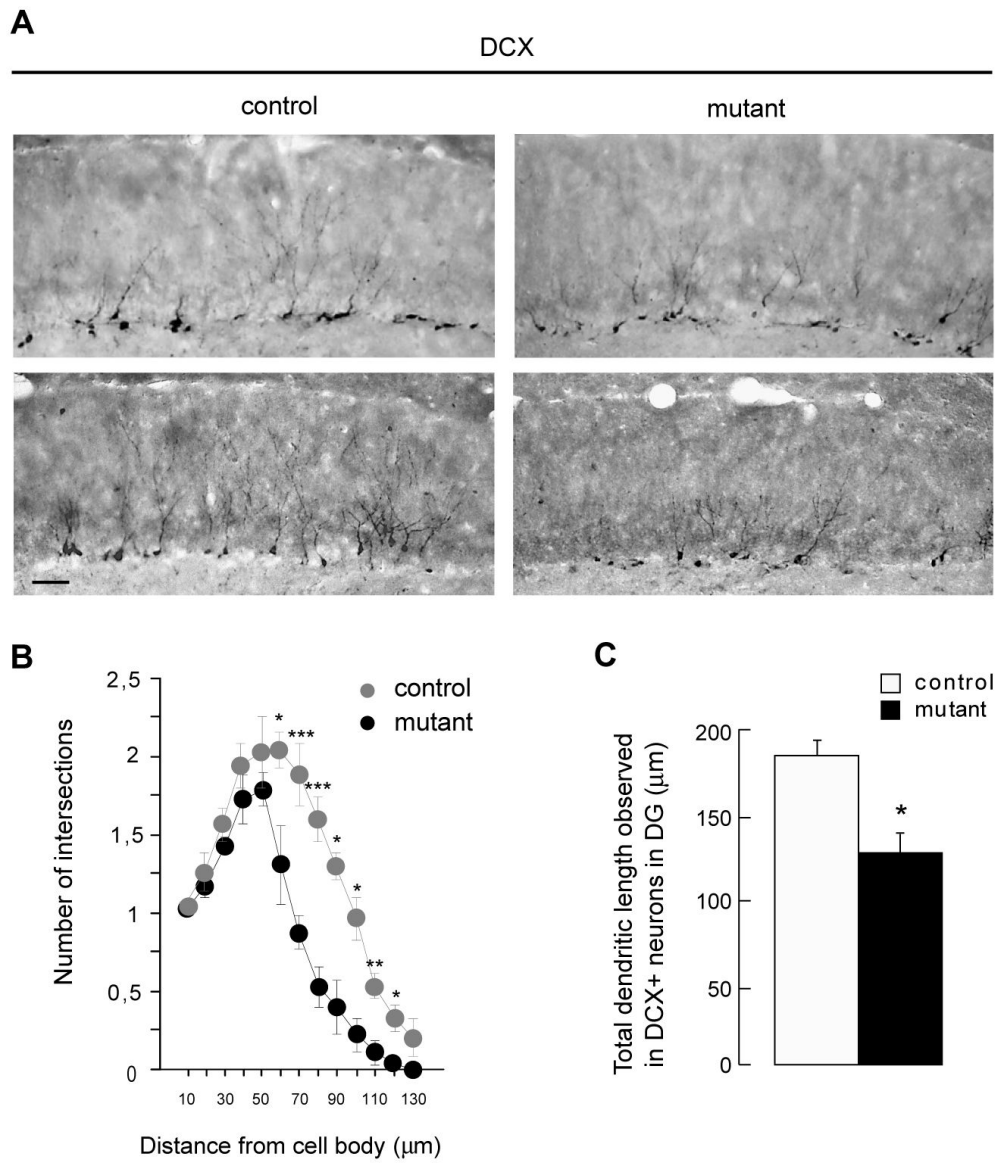
Deletion of huntingtin in mature neurons alters dendritic arborization of newborn hippocampal neurons. (**A**) Representative images of DCX immunostained slices in the upper blade of DG of control and mutant mice 6 months after tamoxifen injection. Scale bar : 50 µm. (**B**) Sholl analysis was performed on 12 randomly chosen DCX positive neurons in DG for each control and mutant mice (n=4 mice per group). (**C**) The total length of the dendrites of 12 randomly chosen DCX positive neurons in DG for each control and mutant mice was measured using NeuroLucida software (n=4 mice per group). * p<0.05 ** p<0.01 *** p<0.005.

To investigate the consequences of HTT depletion further, we analyzed dendritic arborization: the complexity of dendritic arborization is a marker of maturation and differentiation of newly born hippocampal neurons. This marker is altered in knock-in *Htt* ^*Q111/Q111*^ mice [5]. We immunostained brain sections with anti-DCX antibodies (Figure 3A) and performed a Sholl analysis on randomly selected DCX-positive hippocampal neurons to evaluate dendritic arborization in the hippocampus. Mutant mice showed an altered hippocampal dendritic arborization: the numbers of dendrites crossing digital circles of > 60 µm in radius centered on the cell body of neurons were significantly lower in sections from mutant mice than controls (Figure 3B). The total length of dendrites was also shorter in mutant DG (Figure 3C). These findings indicate that the maturation and differentiation of newborn DG neurons is impaired after the specific invalidation of HTT in mature hippocampal and cortical neurons.

### Altered hippocampal BDNF/TrkB signaling in mice deficient for huntingtin in hippocampus and cortex mature neurons

**Figure 4:**
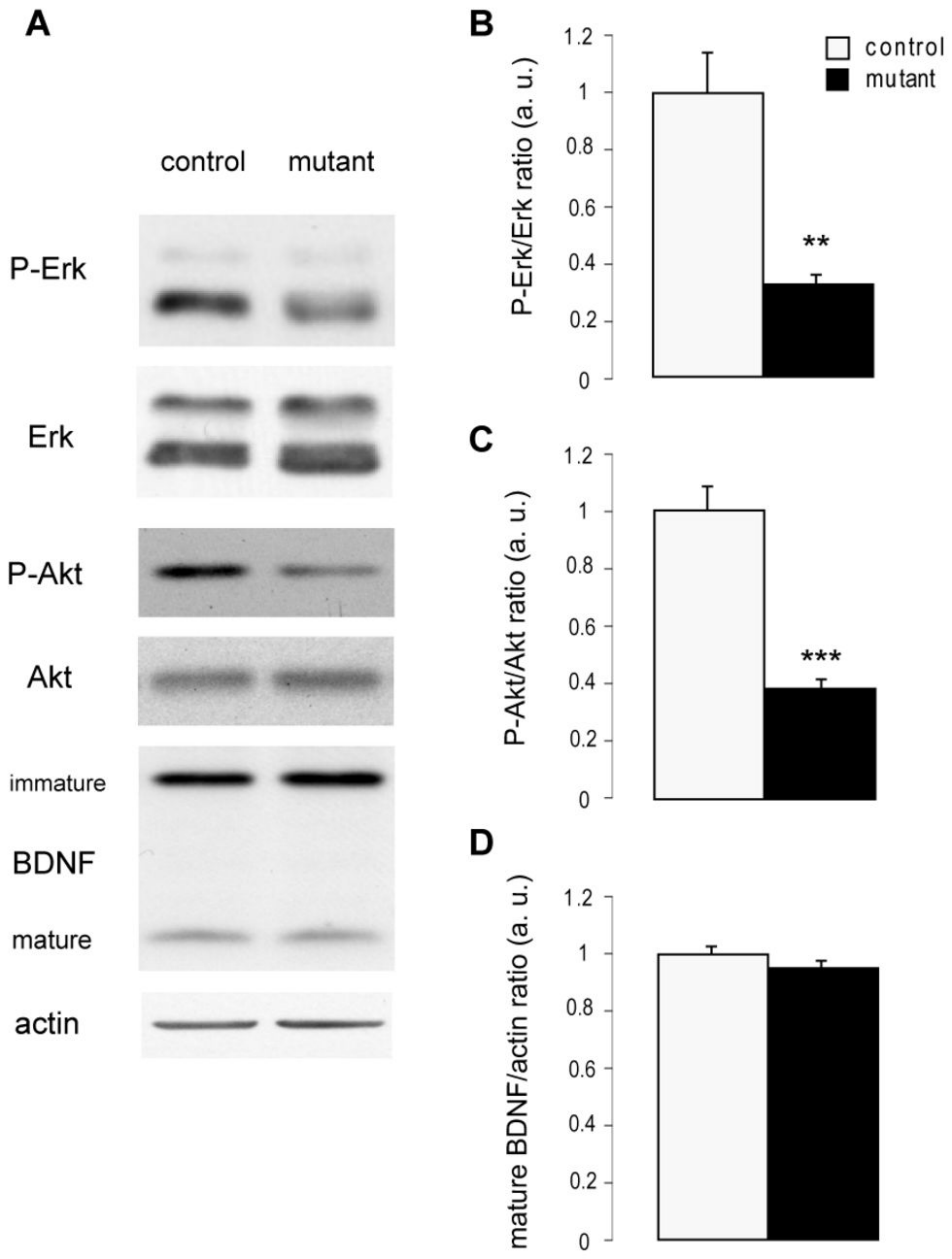
Deletion of huntingtin in mature neurons alters hippocampal Akt and Erk phosphorylation without affecting BDNF production. (**A**) Representative Western-blots of hippocampal proteins extracted from mutant and control mice 6 months after being injected with tamoxifen and incubated with phospho-ERK, ERK, phospho-Akt, Akt, BDNF or actin recognizing antibodies. (**B**–**D**) Data are the mean +/- SEM of the ratios phospho-ERK/ERK, phospho-Akt/Akt, mature BDNF/actin obtained by Western-blot densitometric analysis, normalized so that the mean value of controls is equal to 1 (n= 4-5 per group). * p<0.05 ** p<0.01 *** p<0.005.

The effects observed in newborn hippocampal neurons in mutant mice are strongly reminiscent of the effects following the specific deletion from these neurons of TrkB, the receptor for the neurotrophic factor BDNF [12]. Upon BDNF binding, cell-surface TrkB receptors are activated, leading to the stimulation of downstream kinases, including the extracellular-regulated kinases (Erk1/2) and Akt [13]. To determine whether BDNF/TrkB signaling is altered in the hippocampus of mutant mice, we prepared protein extracts from the hippocampus of mutant and control mice and determined the phosphorylation status of Erk and Akt by Western blotting. Both Erk and Akt were less extensively phosphorylated in mutant mice than their control siblings (Figure 4A–C). We also assayed immature and mature BDNF but found no significant difference between mutants and controls (Figure 4A and 4D). These results suggest that BDNF/TrkB signaling in mutant hippocampus is impaired due to a defect in BDNF transport and release.

**Figure 5:**
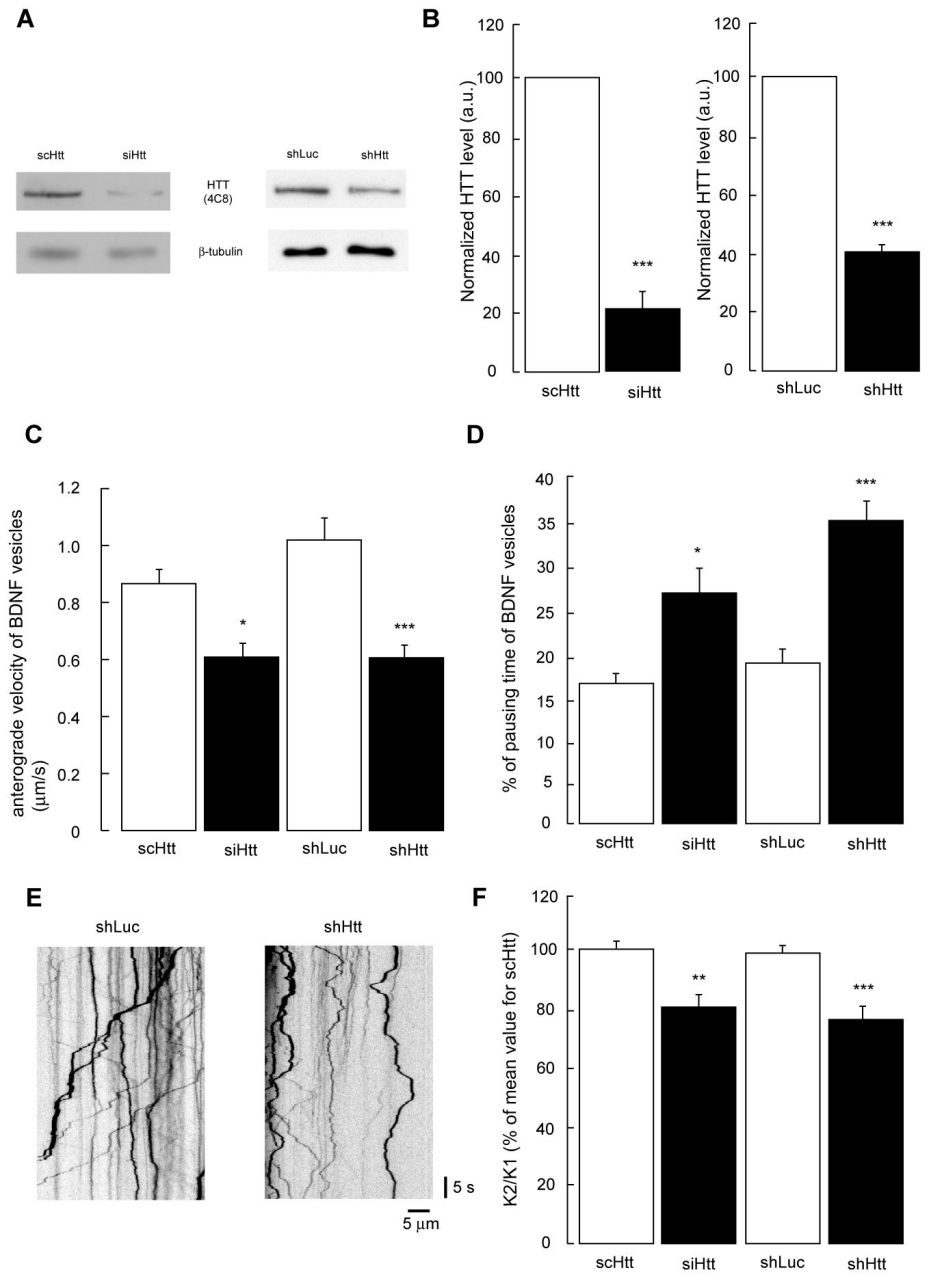
Downregulation of huntingtin expression alters BDNF vesicular trafficking and downregulates BDNF secretion in hippocampal neurons. (**A**,**B**) Rat primary hippocampal neurons were electroporated with BDNF-mCherry and scrambled RNA (scRNA) or siRNA targeting Htt (siHtt) or shRNA targeting luciferase (shLuc) as a control or shRNA targeting Htt (shHtt). Three days later, proteins were extracted and Western-blots were incubated with anti-Htt (4C8) and anti-tubulin antibodies. Data are the mean +/- SEM of 3 independent experiments per group, normalized to the value obtained for the scHtt group or the shLuc group. (**C**, **D**) Three days after electroporation with the constructs described above, the movements of BDNF containing vesicles were observed by videomicroscopy and analyzed with kymographs. Data are the mean +/- SEM of the anterograde velocity (C) or percentage of time a vesicle is pausing (velocity less than 0.05 µm/s) (D) in 18-27 neurons per group analyzed in 3 independent experiments. * p<0.05 ** p<0.01 *** p<0.005 (**E**) Representative kymographs of BDNF-mCherry vesicles in primary hippocampal neurons expressing shLuc or shHtt. (**F**) Rat primary hippocampal neurons were electroporated with BDNF and scrambled RNA (scRNA) or siRNA targeting Htt (siHtt) or shRNA targeting luciferase (shLuc) as a control or shRNA targeting Htt (shHtt). Three days later, a first depolarization allowed the cellular release of BDNF in the medium (K1) and depleted the internal BDNF vesicular store. After 30 min of recovery, a second depolarization was applied and a new sample of the medium was isolated (K2). Concentrations of BDNF in K1 and K2 samples were determined by ELISA. Data are the mean +/- SEM of 3 independent experiments per group, normalized to the value obtained for the scHtt group. * p<0.05 ** p<0.01 *** p<0.005.

Indeed, huntingtin is a major regulator of BDNF transport in cortical neurons, in both physiological and disease conditions, and loss of HTT from cortical neurons reduces BDNF transport [14]. However, whether loss of HTT from hippocampal neurons has an effect in the transport of BDNF has not previously been tested. We therefore downregulated HTT expression in hippocampal neurons in culture using an siRNA targeting *Htt*; this siRNA has been validated and has no off-target effects in neurons as assessed with a rescue strategy [15]. To avoid any possible off-target effects, we also used a shRNA targeting a different region of the coding sequence of *Htt* to reduce HTT levels in hippocampal primary neurons. Neurons were co-electroporated with BDNF-mCherry and with the siRNA or shRNA targeting *Htt*. Scramble RNA and shLuciferase (shLuc) were used as controls, respectively. Both siRNA and shRNA targeting Htt decreased very significantly the level of HTT expressed in neurons, compared to their respective controls (Figure 5A-B).

Using fast videomicroscopy, we measured the dynamics of BDNF-mCherry-containing vesicles as previously reported [15–17]. We observed a statistically significant decrease in the anterograde velocity of BDNF-mCherry vesicles with an increase in their pausing times when HTT in hippocampal neurons was depleted (Figure 5C–E). To further evaluate the physiological relevance of this reduction in the transport of BDNF, we tested whether it affected the capacity of hippocampal neurons to release BDNF. We therefore depolarized hippocampal neurons that were previously electroporated with a human BDNF construct and then assayed BDNF released into the medium after two successive depolarizations. As previously reported, the ratio between the BDNF released after the second to the first depolarization is a measure of the transport-dependent release of BDNF [14,16,18]: the first depolarization releases the pool present at the membrane, and the second release is strictly dependent on microtubule-dependent transport. We found that the transport-dependent release of BDNF by hippocampal neurons was decreased when HTT expression was downregulated either by siRNA or shRNA (Figure 5F). Together, these results indicate that downregulation of HTT in hippocampal neurons leads to a defect in BDNF transport and release in the hippocampus, and a subsequent alteration of the Erk and Akt signaling pathways.

### Mice deleted for huntingtin in the cortex and hippocampus show abnormal anxiety-related behaviors

**Figure 6:**
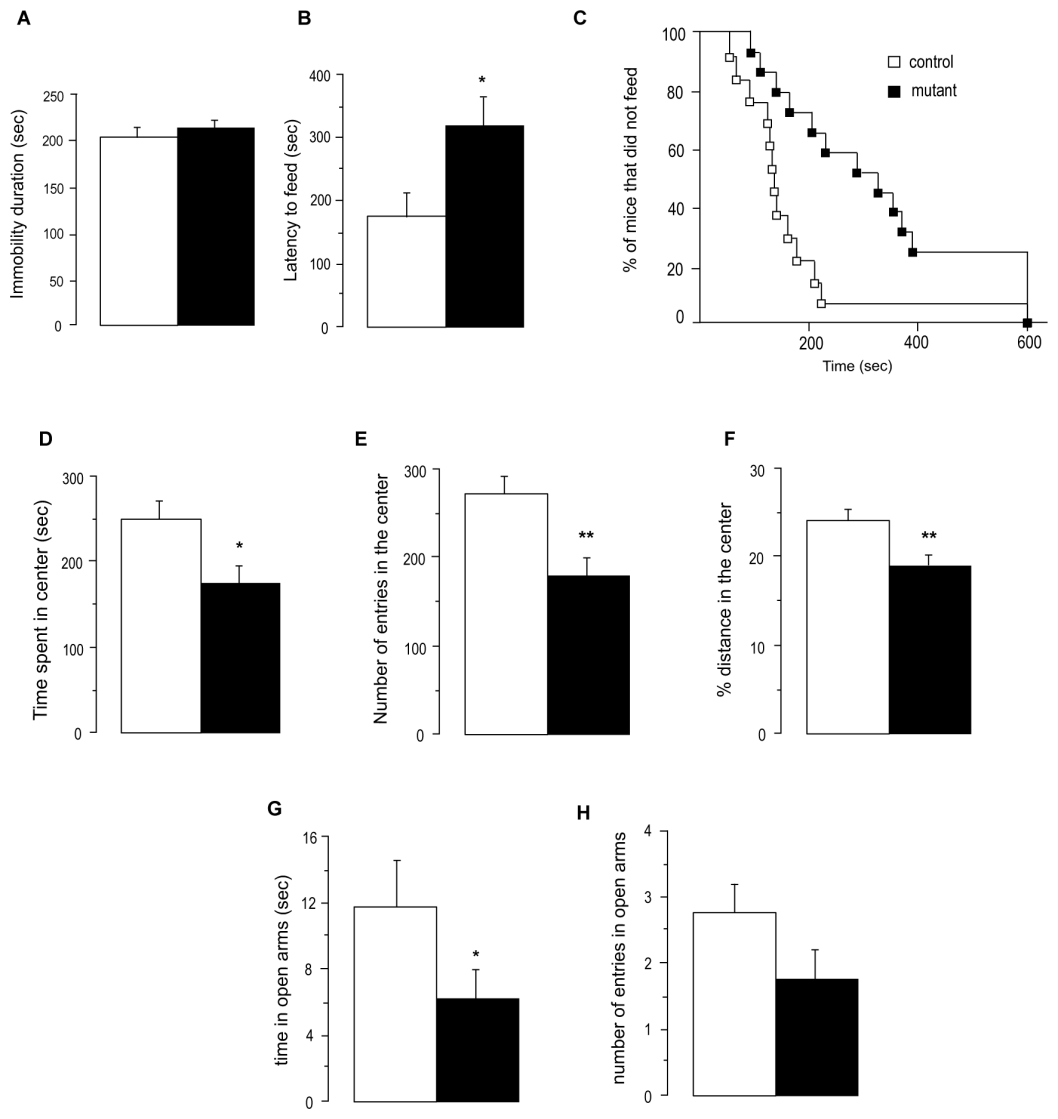
Deletion of huntingtin in mature cortical and hippocampal neurons triggers modifications of anxiety-related behaviors. (**A**) Depressive-like behavior was assessed by the forced swim test (FST) where mice are placed into plastic buckets filled with water and the immobility durations are measured. (**B**–**C**) Mixed anxio-depressive behavior was assessed by the NSF test where starving mice are placed in a corner of a plastic box containing a pellet of food in the center. The latency to feed is timed. The data are presented with means +/- SEM and as a percentage of mice that did not feed per group during the 10 min of the test. (**D**–**E**) Anxiety-related behavior was assessed by placing mice in the center of a Plexiglas open field box, and time and number of entries in the center were measured during 30 minutes. (**F**) In the previous open field test, the distance travelled in the center and the total distance travelled were measured and the percentage of distance travelled in the center over the total distance travelled was calculated. (**G**, **H**) Anxiety-like behaviour was assessed by placing mice in the center of an elevated plus maze (EPM) and time and the number of entries in open arms were measured during 5 minutes. For all the tests, n=12-14 per group. * p<0.05 ** p<0.01 *** p<0.005 for comparisons between genotypes.

Mutant mice 6 months post-tamoxifen treatment were slightly heavier than control mice (Figure S2A). These mice showed no obvious motor deficits in rotarod tests (Figure S2B). We used a depression-related behavioural test : the forced swim test (FST) and found no difference in the immobility behaviour between the two genotypes (Figure 6A). We further investigated anxiety/depression-related behavior using the novelty-suppressed feeding (NSF) paradigm that is sensitive to both anxiety and depressive states. The latency to feeding for mutant mice was significantly longer (Figure 6B-C). This was not due to differences in total food consumption in the home cage between mutants and controls (ANOVA F(1,24)=1.424; p=0.24). Then, we used an open-field (OF) protocol to test anxiety-related behaviors. Mutant mice spent significantly less time than controls in the center of the OF and also entered this area less frequently (Figure 6D-E), suggesting an anxious phenotype. However, mouse locomotor activity in this paradigm was evaluated, and the total ambulatory distance travelled was lower for mutants than controls, although the difference was not significant (ANOVA F(1,24)=3.479; p=0.074). To assess whether the anxious phenotype observed in mutants was a consequence of reduced locomotor behavior, we calculated the distance travelled in the center of the OF as a percentage of the total distance travelled (Figure 6F). This percentage was significantly lower for mutant than control mice, demonstrating that there was an anxiogenic component to the behavior of the mutants, independent of any motor component. Last, we performed the elevated plus maze test (EPM) and found that mutant mice spent significantly less time in open arms than control mice (Figure 6G). The number of entries indicated a tendency of mutant mice to less enter the open arms than the controls (ANOVA F(1,22)=2.752; p=0.11) (Figure 6H).

Overall, our analyses reveal that the depletion of huntingtin in mature cortical and hippocampal neurons has a significant effect on anxiety-related behavior.

## Discussion

We report evidence for a non-cell autonomous mechanism dependent on HTT in the hippocampus: when the mature hippocampal and cortical neurons are depleted of HTT protein, the survival of newborn neurons in the hippocampus is reduced. Indeed, two specific markers, long-term survival and maturation of dendritic arborization, are selectively altered by this non-cell autonomous effect.

Although we observed a tendency for abnormal dendritic arborization of newborn neurons in mutants 3 months after tamoxifen injection, it did not reach significance (data not shown). This defect is significant 6 months after tamoxifen injection, suggesting that neurogenesis defects are age-dependent and/or the consequence of the slow accumulation of cellular defects linked to the absence of HTT. Various models of HD mice have shown progressing phenotypes, with manifestations observed only in aged mice [9–11].

According to [19], most of the newly generated neurons in DG die between 3 days and 28 days after BrdU labeling. Our data showing that survival is affected 42 days but not 21 days after BrdU labeling demonstrate that the non-cell autonomous survival defect of newborn neurons is a late event, either acting at the end of the normal time window and/or as a consequence of a prolonged death period. We did not find any double labeled BrdU/cleaved caspase-3 in the DG of mutant or control mice 42 days after BrdU labeling (data not shown), indicating that the possible prolonged death period has ended at that time. Since the defect in dendritic arborization occurs at around the same stage as reduced neuronal survival was observed, it is plausible that the two events are linked; possibly, newborn neurons that are not branched correctly are more likely to die. Consistent with this possibility, the survival of newborn neurons has been shown to be linked to their functional integration into existing networks [20,21].

Notably, we did not observe defects either in the proliferation of neuron precursors or in mid-term survival. Defects of these types are, however, observed in many HD mouse models [6,10,22]. This discrepancy could be due to polyQ mutated HTT having effects different to those of loss of wild-type HTT. More likely, the defects of proliferation or mid-term survival are associated with cell autonomous roles of HTT in newborn neurons, distinguishable from the non-cell autonomous role of mature neurons on the long-term survival of newborn neurons.

The steps in hippocampal neurogenesis that are defective in *CaMKCreER* ^*T2*^ *; Htt* ^*flox/flox*^ mutants are similar to the steps that have been shown to depend on TrkB signaling in newborn neurons, namely dendritic arborization, and neuronal survival with a similar timeframe [12]. Accordingly, we show that HTT downregulation leads to a defect in the BDNF/TrkB downstream signaling kinases Erk and Akt observed *in vivo*. It was previously reported that Akt and ERK, two well-known prosurvival kinases, also regulate dendritic growth and arborization downstream from BDNF [23]. These findings may provide an explanation for the defective dendritic arborization in the *CaMKCreER* ^*T2*^ *; Htt* ^*flox/flox*^ mutants. We show that the defects in Erk and Akt activation are unlikely to be the consequence of a reduced BDNF production; they are rather the consequence of a reduction in BDNF transport and secretion. Indeed, we found that BDNF vesicle trafficking is impaired when huntingtin is downregulated and that this leads to a decreased capacity of neurons to release BDNF. This effect is consistent with the observation that HTT silencing in cortical neurons results in a decrease in the transport and release of BDNF [14,16]. Moreover, our experimental paradigm of BDNF release suggests that the observed reduced BDNF release is activity dependent. This agrees with previous studies investigating the effect of polyQ expansion on BDNF release [15,18,24,25].

The involvement of HTT in vesicular transport in hippocampal neurons was suggested previously by a study involving overexpression of wild-type and mutant polyQ huntingtin, and APP-YFP as a tag for moving vesicles [26]. Our findings that loss of HTT affects BDNF transport in hippocampal neurons is in agreement with the loss of HTT function in axonal transport in HD: the presence of a polyQ expansion in HTT also results in a decrease in axonal transport in both cortical and hippocampal neurons [14–16,26].

Loss of HTT in mature cortical and hippocampal neurons also enhanced anxiety-like behavior, as assessed by a combination of NSF, OF and EPM tests. There was no effect of the mutation on the FST test, suggesting that a possible depressive-like behavior is not a major component of the phenotype of the mutant mice. The implication of the defect of BDNF release in this phenotype is consistent with results obtained with a Val66Met variant showing an impaired activity-dependent secretion of BDNF [27] and alteration of BDNF/TrkB signaling in hippocampal newborn neurons [12]. NSF results, in particular, have been shown to be dependent on hippocampal neurogenesis [28]. Although we cannot exclude other mechanisms, defect of HTT-dependent BDNF transport and release is likely to contribute to the enhanced anxiety-like phenotype of our mutant. Our findings are supported by the recent study reporting that mice in which the Cdk5 phosphorylation sites are mutated into unphosphorylatable ones show increased BDNF transport and release associated with enhanced hippocampal neurogenesis and a decrease of anxiety-like behaviors [7].

In a previous model, two lines of *Camk2a-Cre* transgenic mice were crossed with *Htt* ^*flox/-*^ mice [9]. In this case the promoter was activated between E15.5 and P5, far earlier than in our model. Also, the transgenic mouse lines used expressed Cre not only in cortex and hippocampus but also in striatum and cerebellum. In contrast to this model, we observed a small but significant weight gain (and not weight loss) in mutants and we did not observe limb clasping upon tail suspension in mutant mice. We did not observe any obvious brain morphological or anatomical abnormalities or an increased TUNEL or cleaved caspase-3 stainings in the brain of mutant mice (data not shown). The apparent discrepancies between the two models can be explained by the fact that in our model the deletion of *Htt* is far more limited in space and, importantly, occurs later when most if not all neuronal connections have been established. It is possible that cortical and hippocampal neurons need HTT during a critical phase in their early life (from P0 to P60) and that the loss of HTT after this period is less crucial for their survival (in a cell autonomous way).

In conclusion, we report here that loss of HTT function in the adult cortex and hippocampus alters neurogenesis and neuronal maturation. We also show that this is probably linked to an alteration of downstream TrkB signaling kinases caused by a downregulation of the axonal transport of BDNF. Loss of HTT function also leads to alteration of anxiety-related behavior. Together with these recent findings [7], our results further suggest that some of the anxiety disorders that are prevalent among HD patients may be caused by an alteration of normal HTT function in the hippocampus and cortex.

## Materials and Methods

### Ethics statement

All experimental procedures were performed in strict accordance with the recommendations of the European Community (86/609/EEC) and the French National Committee (2010/63) for care and use of laboratory animals. Studies were performed in an authorized establishment (Institut Curie, Orsay facility license #C91471108, Feb 2011) under the supervision of authorized investigators (permission #91-589A to P. Pla and permission #92-256B to D.J. David). This study was evaluated and approved by the Comité d’Ethique en matière

d’Expérimentation Animale Paris Centre et Sud (National registration number: #59) presided by Pascal Bigey.

### Mice and tamoxifen injection

*CaMKCreER* ^T2^ mice were obtained from EMMA (European Mouse Mutant Archive, see http://www.emmanet.org/index.php). The generation of these transgenic mice is described in [8]. Their strain of origin is FVB/N.

*CaMKCreER* ^T2^ mice were crossed with *Htt* ^*flox/flox*^ mice [9] to obtain *CaMKCreER* ^*T2*^ *; Hdh* ^*flox/+*^ mice, which were crossed with *Hdh* ^*flox/flox*^ mice to obtain *CaMKCreER* ^*T2*^ *; Hdh* ^*flox/flox*^ mice named as mutants and their littermates *WT; Hdh* ^flox/flox^ mice that were used as controls. Only male mice were used for behavioral, histological and biochemical analyses presented in this article.

Tamoxifen preparation and injection was performed as described in [8]. Briefly, eight-week-old mice were injected intraperitoneally with 1 mg tamoxifen twice a day for five consecutive days.

### Immunohistochemistry

For β-galactosidase whole mount stainings, we followed the same protocol as [8]. To study proliferation mice were administrated with BrdU (150 mg/kg intraperitoneal) 2 hours before sacrifice in control and mutant mice injected 6 months ago with tamoxifen. Alternatively, to study the survival of newborn neurons, BrdU was administrated twice a day during three days 21 or 42 days before sacrifice. After anesthesia with 100 mg/ml ketamine and 20 mg/ml xylazine, mice were perfused transcardially with cold PBS and 4% paraformaldehyde (PFA). The brains were removed, fixed overnight in 4% PFA and then cryoprotected in 30% (w/v) sucrose in PBS before embedding in Tissue-Teck. Serial sections (35 µm) were made on a cryostat. Every sixth floating section was used for each staining, covering the whole hippocampus. Epitopes were unmasked in formamide/SSC (1/1) for 1h at 65°C and in 2N HCl at 37°C for 20 min. After blocking in 5% NGS and 0.1% Triton X-100 in PBS, sections were incubated overnight at 4°C with rat anti-BrdU (1/100), mouse anti-NeuN (1/500) and goat anti-DCX (1/500) antibodies (from Chemicon and Santa Cruz respectively). After washings, sections were incubated at room temperature for 2 hours with secondary biotinylated donkey anti-rat (1/100), chicken anti-mouse A647 (1/200) and donkey anti-goat Cy3 (1/200) secondary antibodies, washed and incubated with streptavidin-Cy2 (1/200) during 1h at room temperature. After washings, sections were mounted in Mowiol-DABCO. For double HTT/GFAP stainings and HTT/DCX stainings, mouse anti-HTT (4C8, Millipore) (1/100), rabbit anti-GFAP (Dako, 1/500) and goat anti-DCX (Santa Cruz, 1/500) antibodies were used, followed by appropriate secondary antibodies at 1/200 in blocking buffer. For the Sholl analysis following DCX immunohistochemistry, 35 µm floating sections were incubated 30 min with 0,3% H_2_0_2_, then blocked 10% foetal bovine serum in 0.1M TrisHCl pH7.4, 0.15M NaCl (=TBS) with 0.5% Triton X100 and incubated overnight at 4°C with goat anti-DCX antibody (1/200) (Santa Cruz) in blocking solution. After washes in 0.1M TBS, sections were incubated in biotinylated bovine anti-goat antibody (1/500) and procedure was finished using the ABC kit and DAB staining procedure from Vector Laboratories. Sections were mounted on slides, dehydrated in ethanol, delipidated in xylene and mounted with Entellan.

Sholl analysis was performed on 12 randomly chosen DCX positive hippocampal neurons per brain that presented at least a second order dendrite, using NeuroLucida software (MicroBrightField, Williston, VT) on an Olympus BX51 microscope equipped with a motorized stage device and x100 immersion oil objective. All samples were number coded, and analysis was done blind to treatment.

### Western blotting analysis

For Western Blot analysis, mice were killed by cervical dislocation, hippocampi were rapidly dissected and the proteins were extracted in 20 mM Tris-HCl pH7,6, 137 mM NaCl, 1% NP-40, 10% glycerol, 1% protease and phosphatase inhibitor cocktails (Sigma). Cell lysates were centrifuged 10,000g for 10 min at 4°C. Electrophoresis was performed in 8% (or 15% for BDNF detection) SDS-polyacrylamide gels and the proteins were transferred to Protran nitrocellulose membranes (Whatman). After blocking in 10 mM Tris-HCl pH7.6, 200 mM NaCl, 0,1% Tween-20 and 5% BSA, membranes were incubated with anti-phospho ERK (rabbit polyclonal 9101), anti-ERK (rabbit polyclonal 9102), anti-phospho-Akt (rabbit monoclonal 4058), and anti-Akt (rabbit polyclonal 9272) antibodies to detect phosphorylated and total ERK and phosphorylated and total Akt, respectively. All antibodies were purchased from Cell Signaling Technology. Mature and precursor BDNF were detected with an anti-BDNF antibody (rabbit polyclonal sc-546, Santa Cruz). All antibodies were diluted 1/1000 in blocking buffer. Huntingtin was detected with 4C8 antibody (mouse monoclonal MAB2166, Millipore) diluted 1/5000 in blocking buffer. Primary antibodies were detected with appropriate HRP-coupled secondary antibodies and ECL signals (SuperSignal West Pico Chemiluminescent Substrate (Pierce, Erembodegem, Belgium) were quantitated with image analysis (ImageJ).

### BDNF vesicular trafficking and secretion

To analyze BDNF vesicular trafficking, E17 rat primary hippocampal neurons were prepared as previously described [14]. Five million neurons were electroporated in Amaxa Nucleofactor Kit for Rat Neurons (Lonza) with 2 µg of BDNF-Cherry and 1 µg of siRNA or 2 µg of shRNA constructs and plated on glass coverslips coated with poly-L-lysine (1 mg/mL) in Neurobasal supplemented 2% B27, 2 mM GlutaMax, 1% penicillin/streptomycin, 10 µM forskolin and 100 µM IBMX. The plasmid BDNF-mCherry was a generous gift from G. Banker (Oregon Health and Science University, Portland, Oregon). siRNA targeting huntingtin had the following sense sequence : GGAACUCUCAGCCACCAAGTT and scRNA had the following sense sequence : AUCGAGCUACCACGAACGCTT and both were synthesized and annealed with their antisense sequence by Eurogentec. They have been previously used and tested in [15]. shRNA contracts SIN-PGK-GFP-WHV-LTR-TRE-siHtt6 (shHtt) and the control SIN-PGK-GFP-WHV-LTR-H1-siLuc (shLuc) were a generous gift from N. Déglon (Centre Hospitalier Universitaire Vaudois, Lausanne, Switzerland) and described and validated in [29].

Videomicroscopy experiments were performed 3 days after transfection. Glass coverslips with neurons were mounted in a Ludin chamber. The microscope and the chamber were kept at 37°C. Images were recorded with a 100X PlanApo N.A. 1.4 oil immersion objective on a Leica DM IRBE microscope and collected every 200 milliseconds in stream mode with 150 milliseconds of exposure and a 2 x 2 binning using a Micromax camera (Ropper scientific, Trenton, NJ, USA) controlled by Metamorph software (Molecular Devices, Sunnyvale, CA, USA). Videos, kymographs, and analyses were generated with ImageJ software (http://rsb.info.nih.gov/ij/, NIH, USA) and the KymoToolbox plug-in [30].

The analysis of BDNF transport-dependent secretion was done as described in [15]. Briefly, hippocampal neurons were electroporated with a non-tagged version of human BDNF. Three days later, neurons were depolarized (treatment for 20 min with neuronal culture media containing 28 mM KCl, 30 mM CaCl_2_, 30 mM NaCl). The supernatant was recovered (K1 fraction). The neurons were washed and incubated 30 min in normal neuronal culture medium and depolarized again. The supernatant K2 was recovered and a whole protein lysate (L) of cells was extracted to assess the general expression of BDNF. BDNF levels were measured by anti-BDNF sandwich-ELISA using BDNF E_max_ Immunoassay system (Promega, France), following manufacturer’s instructions.

### Behavioral analyses

Motor performance was assessed using a rotarod. Mice were placed on the rod, and the number of falls in the subsequent 3 minutes was counted.

Depression-related behavior was assessed in the forced swim test (FST). Mice were placed into plastic buckets (19 cm diameter, 30 cm depth, filled with 23–25°C water) and videotaped from the top for the 6 min session. Only the last 4 min were scored using X’PERT FST (Bioseb, France) for immobility duration.

Novelty suppressed feeding (NSF) test is a conflict test with competing motivations: the drive to eat and the fear of venturing into the center of a brightly lit arena. The testing apparatus consisted in a plastic box (50 cm × 30 cm × 15 cm), the floor of which was covered with wooden bedding. 24 h prior to testing, all food was removed from the home cage. At the time of testing, a single pellet of food was placed on a white paper in the center of the box. The animal was placed in a corner of the box, and a stopwatch was immediately started. The latency to eat (defined as the mouse sitting on its haunches and biting the pellet with the use of forepaws) was timed. Immediately afterwards, the animal was transferred to its home cage, and the amount of food consumed in the subsequent 5 min was measured.

Anxiety-related behavior was measured using an open field (OF). Anxiety parameters and motor activity were quantified during 30 min in Plexiglas open field boxes as previously described in [28]. Anxiety-related behavior was also tested using an elevated plus maze (EPM) : after placing a mouse in the central platform of the EPM, facing a closed arm, and videotaping the exploration of the mouse, time in open arms and the number of entries in open arms were measured by Any-maze software (Stoelting Inc.).

### Statistical analyses

Quantifications were performed by an experimenter blind to mouse genotype. Statview 4.5 software (SAS Institute Inc., Cary, NC) was used for statistical analysis. Data are expressed as mean +/- S.E.M. They were analyzed using a one-way ANOVA using genotype as a factor. The criterion for statistical significance was set at p<0.05.

## Supporting Information

Figure S1 **Total amount of DCX+ DG neurons is not affected by the deletion of huntingtin in mature neurons.** Floating slices of brains from control and mutant mice injected with tamoxifen 6 months prior processing were treated for immunohistochemistry with anti-DCX antibody and appropriate secondary antibody and treatments for DAB staining. The number of DCX^+^ neurons in DG was scored (n=3-4 per group).
(TIF)

Figure S2 **Motor performance on rotarod is not affected 6 months after deletion of huntingtin in mature neurons.** (**A**) The weight of control and mutant mice was assessed 6 months after tamoxifen injection. (**B**) Motor performance was assessed by placing control and mutant mice 6 months after tamoxifen injection on a rotarod, and the number of falls in the subsequent 3 minutes was counted (n=12-14 per group).
(TIF)
