# Supplementary figures and images for: Huntingtin Acts Non Cell-Autonomously on Hippocampal Neurogenesis and Controls Anxiety-Related Behaviors in Adult Mouse
Source: PLoS One. 2013 Sep 3;8(9):e73902. doi: 10.1371/journal.pone.0073902 (PMC3760801; doi:10.1371/journal.pone.0073902)

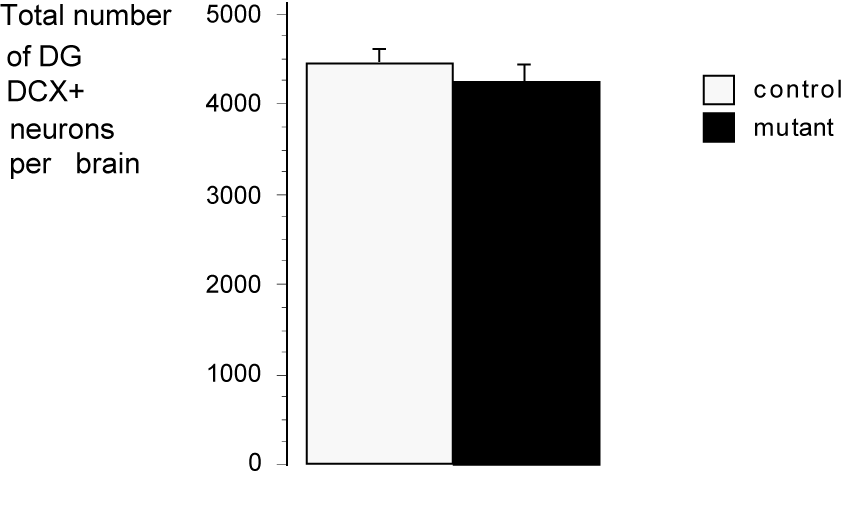

Supplement: Figure S1 — Total amount of DCX+ DG neurons is not affected by the deletion of huntingtin in mature neurons. Floating slices of brains from control and mutant mice injected with tamoxifen 6 months prior processing were treated for immunohistochemistry with anti-DCX antibody and appropriate secondary antibody and treatments for DAB staining. The number of DCX+ neurons in DG was scored (n=3-4 per group). (TIF) [file pone.0073902.s001.tif]

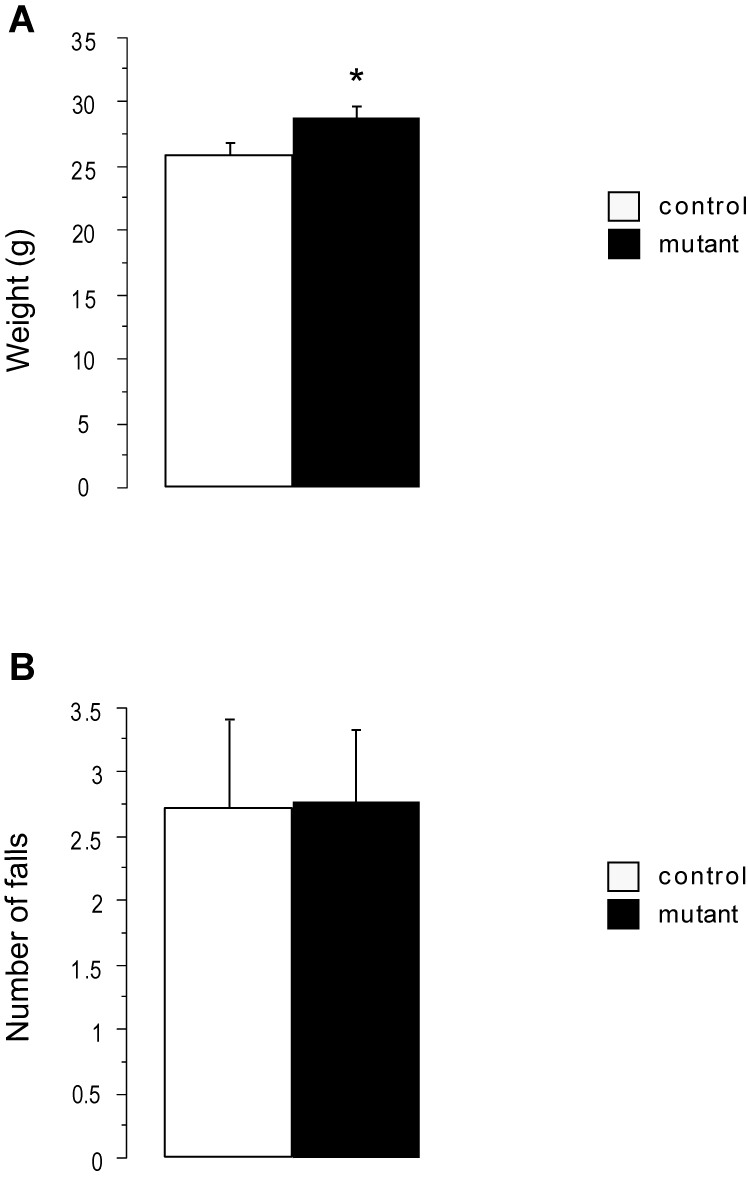

Supplement: Figure S2 — Motor performance on rotarod is not affected 6 months after deletion of huntingtin in mature neurons. (A) The weight of control and mutant mice was assessed 6 months after tamoxifen injection. (B) Motor performance was assessed by placing control and mutant mice 6 months after tamoxifen injection on a rotarod, and the number of falls in the subsequent 3 minutes was counted (n=12-14 per group). (TIF) [file pone.0073902.s002.tif]
